# Comprehensive Characterization of the Vascular Effects of Cisplatin-Based Chemotherapy in Patients With Testicular Cancer

**DOI:** 10.1016/j.jaccao.2020.06.004

**Published:** 2020-09-15

**Authors:** Alan C. Cameron, Kelly McMahon, Mark Hall, Karla B. Neves, Francisco J. Rios, Augusto C. Montezano, Paul Welsh, Ashita Waterston, Jeff White, Patrick B. Mark, Rhian M. Touyz, Ninian N. Lang

**Affiliations:** a BHF Glasgow Cardiovascular Research Centre, Institute of Cardiovascular and Medical Sciences, University of Glasgow, Glasgow, United Kingdom; b McGill University Health Centre, McGill University, Montreal, Quebec, Canada; c Department of Medical Oncology, Beatson West of Scotland Cancer Centre, Glasgow, United Kingdom

**Keywords:** germ cell tumors, platinum therapy, testicular cancer, thrombosis, ACh, acetylcholine, BEP, bleomycin, etoposide and cisplatin, BK, bradykinin, FBF, forearm blood flow, 0FMD, flow-mediated dilatation, ICAM, intracellular adhesion molecule, PAI, plasminogen activator inhibitor, SNP, sodium nitroprusside, t-PA, tissue plasminogen activator, vWF, von Willebrand factor

## Abstract

**Background:**

Cisplatin-based chemotherapy increases the risk of cardiovascular and renal disease.

**Objectives:**

We aimed to define the time course, pathophysiology, and approaches to prevent cardiovascular disease associated with cisplatin-based chemotherapy.

**Methods:**

Two cohorts of patients with a history of testicular cancer (n = 53) were recruited. Cohort 1 consisted of 27 men undergoing treatment with: 1) surveillance; 2) 1 to 2 cycles of bleomycin, etoposide, and cisplatin (BEP) chemotherapy (low-intensity cisplatin); or 3) 3 to 4 cycles of BEP (high-intensity cisplatin). Endothelial function (percentage flow-mediated dilatation) and cardiovascular biomarkers were assessed at 6 visits over 9 months. Cohort 2 consisted of 26 men previously treated 1 to 7 years ago with surveillance or 3 to 4 cycles BEP. Vasomotor and fibrinolytic responses to bradykinin, acetylcholine, and sodium nitroprusside were evaluated using forearm venous occlusion plethysmography.

**Results:**

In cohort 1, the percentage flow-mediated dilatation decreased 24 h after the first cisplatin dose in patients managed with 3 to 4 cycles BEP (10.9 ± 0.9 vs. 16.7 ± 1.6; p < 0.01) but was unchanged from baseline thereafter. Six weeks after starting 3 to 4 cycles BEP, there were increased serum cholesterol levels (7.2 ± 0.5 mmol/l vs. 5.5 ± 0.2 mmol/l; p = 0.01), hemoglobin A1c (41.8 ± 2.0 mmol/l vs. 35.5 ± 1.2 mmol/l; p < 0.001), von Willebrand factor antigen (62.4 ± 5.4 mmol/l vs. 45.2 ± 2.8 mmol/l; p = 0.048) and cystatin C (0.91 ± 0.07 mmol/l vs. 0.65 ± 0.09 mmol/l; p < 0.01). In cohort 2, intra-arterial bradykinin, acetylcholine, and sodium nitroprusside caused dose-dependent vasodilation (p < 0.0001). Vasomotor responses, endogenous fibrinolytic factor release, and cardiovascular biomarkers were not different in patients managed with 3 to 4 cycles of BEP versus surveillance.

**Conclusions:**

Cisplatin-based chemotherapy induces acute and transient endothelial dysfunction, dyslipidemia, hyperglycemia, and nephrotoxicity in the early phases of treatment. Cardiovascular and renal protective strategies should target the early perichemotherapy period. (Clinical Characterisation of the Vascular Effects of Cis-platinum Based Chemotherapy in Patients With Testicular Cancer [VECTOR], NCT03557177; Intermediate and Long Term Vascular Effects of Cisplatin in Patients With Testicular Cancer [INTELLECT], NCT03557164)

Testicular cancer is the most common malignancy in men aged 20 to 40 years, and cisplatin-based chemotherapy with bleomycin, etoposide, and cisplatin (BEP) achieves a cure for almost all patients (1,2). However, this treatment is associated with cardiovascular disease (CVD), including myocardial infarction, thrombosis, and nephrotoxicity (3,4). Adverse effects on the endothelium appear to be a central pathophysiological mechanism (5), and perturbations in metabolic and inflammatory parameters may also be important (6,7). A retrospective epidemiological study of >15,000 patients demonstrated a 5-fold increase in standardized cardiovascular mortality following cisplatin-based chemotherapy that was confined primarily to the first year following treatment (1). This challenges the preconception that cardiovascular risk in testicular cancer survivors is a late phenomenon (3,8).

Historically, the prospective evaluation of vascular effects of cisplatin-based chemotherapy has been hindered by assessments in heterogeneous groups with differing cardiovascular risk, cancer types, and treatment regimens. Furthermore, examination of immediate effects has been limited and usually without longitudinal assessment (9, 10, 11). Understanding the time course and pathophysiological basis of cisplatin-induced vascular and renal injury is critical to inform surveillance and trials of treatment and prevention strategies.

Thus, we assessed the effects of cisplatin-based chemotherapy on endothelial function, metabolic parameters, fibrinolytic factors, and cardiovascular and renal biomarkers in the immediate peritreatment phase followed by prospective, longitudinal assessments over 9 months in men with testicular cancer. In a further series of studies, we used forearm venous occlusion plethysmography, the gold standard method to evaluate endothelial function (12), to assess endothelial vasomotor and endogenous fibrinolytic function in testicular cancer survivors treated 1 to 7 years prior. We also assessed the in vitro effects of cisplatin on stress kinase signaling and thrombosis pathways in human aortic endothelial cell (HAEC) culture.

## Methods

The studies (NCT03557177 and NCT03557164) were approved by the West of Scotland Research Ethics Committee 4 and conducted in accordance with the Declaration of Helsinki. Written informed consent was obtained from all participants.

### Cohort 1: Early effects of cisplatin-based chemotherapy

#### Study participants

Patients were recruited from the Beatson West of Scotland Cancer Centre between January 2016 and July 2017. Inclusion criteria included diagnosis of testicular/retroperitoneal germ cell cancer with orchidectomy ≤8 weeks prior and scheduled for cisplatin-based chemotherapy or surveillance. Participants were categorized into 3 groups: 1) surveillance; 2) 1 to 2 cycles BEP; or 3) 3 to 4 cycles BEP. Exclusion criteria included: carboplatin treatment; age <18 or >65 years; clinical trial participation; antiplatelet/lipid-lowering therapy; recreational drug use; inflammatory/infective/autoimmune disease; another malignancy in the previous 5 years; previous thrombosis; and inability to provide informed consent.

#### Chemotherapy regimens

Cisplatin-based chemotherapy regimens included BEP or etoposide and cisplatin (EP). Each treatment cycle lasted 21 days, with cisplatin administered on days 1 and 2 (cisplatin dose 50 mg/m^2^/day) or days 1 to 5 (cisplatin dose 20 mg/m^2^/day), such that the cumulative dose of cisplatin in each cycle of treatment was 100 mg/m^2^. Patients attended outpatient bleomycin administration on days 8 and 15 (each dose 30,000 IU). Patients were treated with etoposide 165 mg/m^2^ on days 1 to 3 if receiving 1, 3, or 4 cycles of BEP; or etoposide 120 mg/m^2^ on days 1 to 3 if receiving 2 cycles of BEP. Patients with stage 1 disease received 1 to 2 cycles of BEP, a low-intensity cisplatin regimen. Patients with metastatic disease received 3 or 4 cycles of BEP, a high-intensity cisplatin regimen. Patients with metastatic disease or with a contraindication to bleomycin received 4 cycles of EP, with cisplatin administered over days 1 to 5 (cisplatin dose 20 mg/m^2^/day) to achieve cumulative cisplatin dose 100 mg/m^2^. All patients received hydration containing potassium and magnesium before and after each cisplatin dose.

#### Study assessments

**Figure 1:**
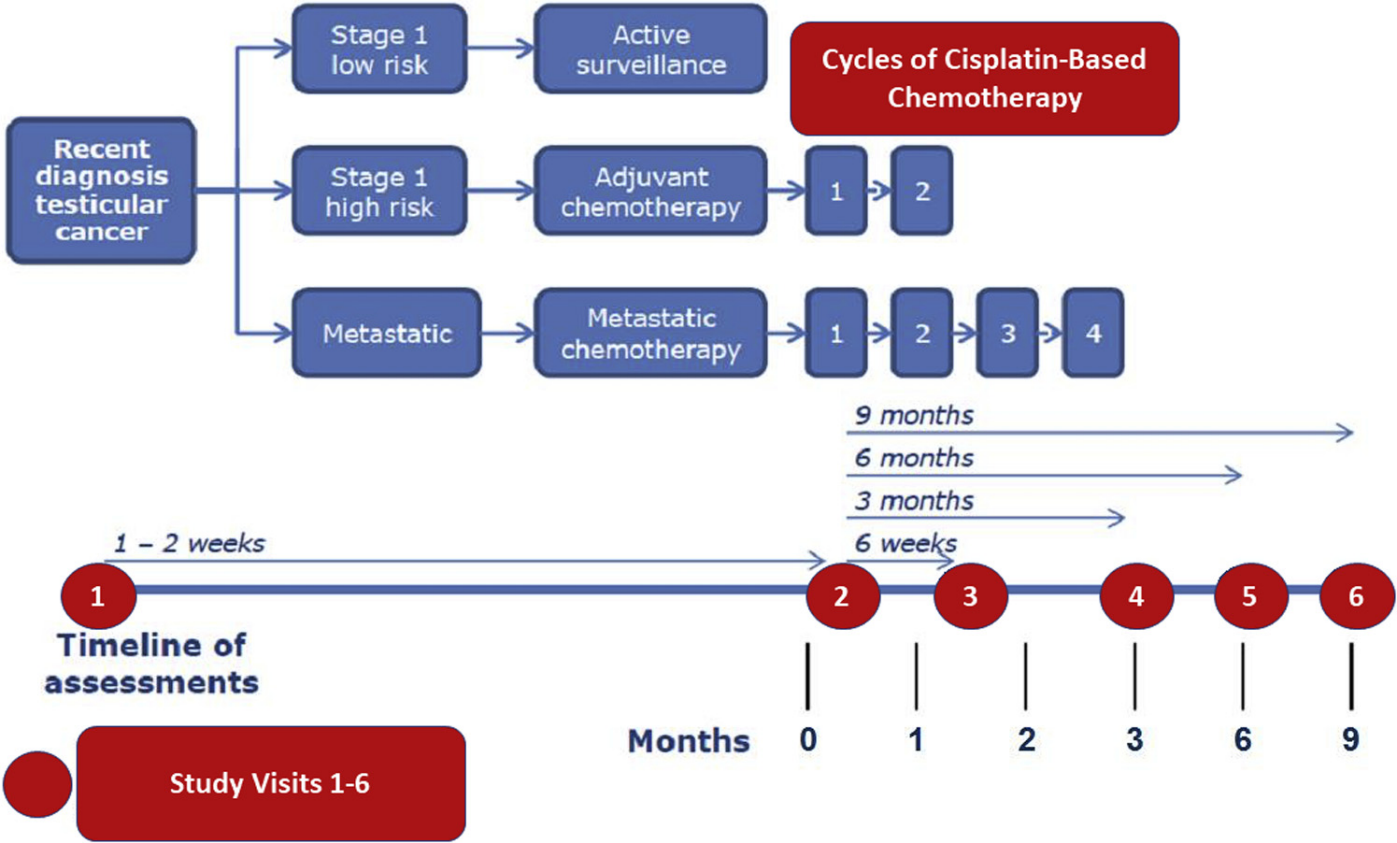
Cancer Management Regimes and Assessments in Cohort 1 (Early Effects Study) Patients with a recent diagnosis of testicular/retroperitoneal germ cell cancer and orchidectomy ≤8 weeks prior scheduled for cisplatin-based chemotherapy or active surveillance were recruited. Participants were stratified into 3 groups by management: 1) active surveillance for stage 1 low-risk disease; 2) 1 to 2 cycles of adjuvant bleomycin, etoposide, and cisplatin (BEP) chemotherapy for stage 1 high-risk disease; or 3) 3 to 4 cycles BEP chemotherapy for metastatic disease. **Red circles** illustrate study assessments.

Initial assessments were performed ≤8 weeks after orchidectomy and ≤2 weeks pre-chemotherapy. In patients managed with surveillance, subsequent assessments were 1 to 2 weeks, 6 weeks ± 3 days, 3 months ± 1 week, 6 months ± 1 week, and 9 months ± 1 week after the initial assessment. In patients managed with chemotherapy, subsequent assessments were within 24 h of cisplatin administration and 6 weeks ± 3 days, 3 months ± 1 week, 6 months ± 1 week and 9 months ± 1 week (Figure 1). Participants fasted (with the exception of water) for 8 h and abstained from exercise, caffeine, and tobacco for 4 h before each assessment. Height, weight, and blood pressure (BP) were assessed at each visit.

#### Primary outcome: endothelial function

The primary outcome was change in endothelial function relative to baseline, assessed using the AngioDefender system (Everist Genomics, Ann Arbor, Michigan). This portable device allows bedside assessment of endothelial vasomotor function. It calculates percentage flow-mediated dilatation (%FMD) using a proprietary algorithm deriving changes in brachial artery diameter from pulse wave amplitude data before and after brachial artery occlusion with an upper arm cuff (13). Maximal post-occlusion change in brachial artery diameter relative to baseline is calculated and expressed as %FMD. The repeatability of AngioDefender %FMD is similar to traditional brachial artery ultrasound (BAUSS) assessment of %FMD (coefficient of variation 25.9% and 25.1%, respectively, according to personal communication with investigative teams (Everist Health, March 2015) for NCT02641197 and NCT02682576. AngioDefender quantification of %FMD is similar to BAUSS (Pearson’s correlation coefficient, r_p_ = 0.75; p < 0.0001) and correlates more strongly with 10-year Framingham risk score (r_p_ = −0.38; p < 0.001 AngioDefender; r_p_ = −0.24; p = 0.03 BAUSS) (14).

#### Secondary outcomes: cardiovascular and renal biomarkers

Fasting venous blood was collected at each visit. Serum creatinine, lipid profile, glycated hemoglobin A1c (HbA1C), and urine albumin to creatinine ratio (ACR) were measured in the local clinical laboratory. Estimated glomerular filtration rate (eGFR) was calculated using the 2009 Chronic Kidney Disease Epidemiology Collaboration creatinine equation (15). Enzyme-linked immunosorbent assays were performed to measure serum tissue plasminogen activator (Asserachrom, Stago, Reading, United Kingdom), plasminogen activator inhibitor-1 (Asserachrom), von Willebrand factor (vWF) (Asserachrom), and intracellular adhesion molecule (ICAM)-1 (Quantikine, R&D Systems, Abingdon, United Kingdom). Cystatin-C was measured using a particle enhanced turbidimetric immunoassay (Tina-quant, Roche, Germany). Serum high-sensitivity C-reactive protein and lipoprotein(a) were measured (Roche c311 analyzer) and high-sensitivity troponin-I was measured (Abbot, Architect i1000SR). Urine was collected for assessment of interleukin (IL)-18 (Quantikine, R&D Systems).

### Cohort 2: Medium-term effects of cisplatin-based chemotherapy

#### Participants

Testicular cancer survivors age 18 to 50 years managed with 3 to 4 cycles of BEP or active surveillance 1 to 7 years previously were recruited. Exclusion criteria included ongoing clinical trial participation; vascular disease; asthma; chronic obstructive pulmonary disease; diabetes; atrial fibrillation/flutter; anticoagulation; tobacco/recreational drug use; inflammatory, infectious, or autoimmune disease; another malignancy within 7 years; and prior thrombosis.

#### Forearm venous occlusion plethysmography

Studies were performed with the patient supine in a quiet, temperature-controlled room. Participants fasted for 4 h, abstained from alcohol for 24 h, and did not consume medications for 3 days before each study. Bilateral venous cannulae were inserted into large antecubital fossa veins for venous sampling. Brachial artery cannulation was performed using a 27-standard-wire-gauge steel needle for intra-arterial administration of locally active doses of drugs. Forearm blood flow (FBF) was measured in the infused and noninfused arms by venous occlusion plethysmography. Supine heart rate (HR) and BP were monitored (12,16).

#### Pharmaceutical agents

Pharmaceutical-grade bradykinin (BK) (Bachem, Bubendorf, Switzerland), acetylcholine (ACh) (Novartis Pharmaceuticals, London, United Kingdom), and sodium nitroprusside (SNP) (UL Medicines, Surrey, United Kingdom) were dissolved in physiological saline. BK is an endothelium-dependent vasodilator that provokes endothelial release of tissue plasminogen activator (t-PA). ACh is an endothelium-dependent vasodilator that does not provoke t-PA release. SNP is an endothelium-independent vasodilator.

#### Outcomes

The primary outcome was change in BK-induced vasodilation. Secondary outcomes were change in ACh- and SNP-induced vasodilation, BK-induced tPA and plasminogen activator inhibitor (PAI)-1 release, and between-group differences in cardiovascular biomarkers.

#### Intra-arterial drug administration

After a 20-min intra-arterial 0.9% saline infusion, participants received ascending doses of BK (100, 300, and 1,000 pmol/min), ACh (5, 10, and 20 μg/min), and SNP (2, 4, and 8 μg/min) for 6 min at each dose, with a 20-min 0.9% saline washout between agents. The infusion rate was maintained at 1 ml/min and the infusion order was randomized for each volunteer.

#### Blood sampling

Venous blood was collected at baseline for lipid profile, HbA1C, vWF antigen, and ICAM-1 concentration (Vacuette, Kremsmünster, Austria). Full blood count, renal function, liver function, lipid profile, glucose, and HbA1C concentrations were measured in local clinical laboratories. Blood samples were simultaneously drawn from each arm at the end of equilibration and each BK dose into acidified buffered citrate (TriniLIZE Stabilyte, Co., Wicklow, Ireland) for t-PA assays and citrate (Vacuette) for analysis of PAI-1 (the major endogenous inhibitor of t-PA). Enzyme-linked immunosorbent assays were performed as described in the previous text to determine concentrations of t-PA antigen, PAI-1 antigen, vWF and ICAM-1, and PAI-1 activity (2B Scientific, Oxfordshire, United Kingdom).

### Human aortic endothelial cells

HAECs (Life Technologies, Paisley, United Kingdom) were cultured in endothelial cell growth medium (Promocell, Heidelberg, Germany) supplemented with 15 ml SupplementMix (Promocell) and penicillin/streptomycin 50 μg/ml. Confluent cells were rendered quiescent by serum starvation for 2 h in low-serum medium with 0.5% fetal bovine serum. Cells were stimulated with cisplatin (1, 3, or 15 μg/ml) (Accord Healthcare, Devon, United Kingdom) or vehicle (phosphate-buffered saline) for 5 min, 15 min, and 24 h.

#### Immunoblotting

HAECs were homogenized in lysis buffer and proteins (30 μg) were separated by electrophoresis as described previously (17). Membranes were probed with antiphosphorylated Akt (Cell Signalling [Danvers, Massachusetts] 4060, 1:1,000) and antiphosphorylated extracellular signal-regulated kinases 1/2 (ERK 1/2) (Cell Signalling 9101, 1:1,000). Protein phosphorylation levels were normalized to α-tubulin (Abcam ab4074, 1:1,0000) and expressed as percentage of the respective time point control, which was taken as 100%.

#### Quantitative real-time polymerase chain reaction

mRNA expression of t-PA (QT00075761, Qiagen, Manchester, United Kingdom) and PAI-1 (QT00062496, Qiagen) was assessed by qPCR. Total RNA was extracted using TRIzol (Qiagen) as previously described (17). Data are expressed as target gene/GAPDH housekeeping gene (Sense: GAGTCAACGGATTTGGTCGT; Anti-Sense: TTGATTTTGGAGGGATCTCG; Eurofins Genomics, Ebersberg, Germany). Relative gene expression was calculated by the 2^-ΔΔCt^ method, and results were compared with control.

### Data analysis and statistics

#### Cohort 1: early effects of cisplatin-based chemotherapy

Data were analyzed using 2-way analysis of variance (ANOVA) with repeated measures based on the general linear model and Dunnett’s correction for multiple comparisons. Power calculations determined that, at a significance of 5%, 10 subjects/group would provide 90% power of detecting 1.6% difference in %FMD between visits by paired Student’s *t*-test with SD of paired difference of 1.4 (13). Serum lipoprotein(a), urine ACR, and urine IL-18 (adjusted for urine creatinine concentration) were logarithmically transformed to ensure normality.

#### Cohort 2: medium-term effects of cisplatin-based chemotherapy

Forearm plethysmographic data were analyzed as described previously (18). Net t-PA and PAI-1 release were defined as the product of the infused forearm plasma flow and the concentration difference between infused and noninfused arms (18). Previous studies demonstrated that 8 subjects per group provides sufficient power to detect an approximately 20% change in FBF at 5% significance (19,20). The influence of a range of factors on FBF responses have been reported in similar sample sizes (21, 22, 23, 24). Analysis was by repeated measures based on the general linear model or 1-way ANOVA.

### Endothelial cell culture

Data were analyzed by 1-way ANOVA with Dunnett’s correction for multiple comparisons (Western Blot analyses) or unpaired Student’s *t*-test (mRNA expression). Relative mRNA expression values were logarithmically transformed to ensure normality.

Variables are reported as mean ± SEM. Statistical analyses were performed using GraphPad Prism (GraphPad Software, La Jolla, California) with statistical significance at 5%.

## Results

### Cohort 1: Early effects of cisplatin-based chemotherapy

#### Vascular function

**Figure 2:**
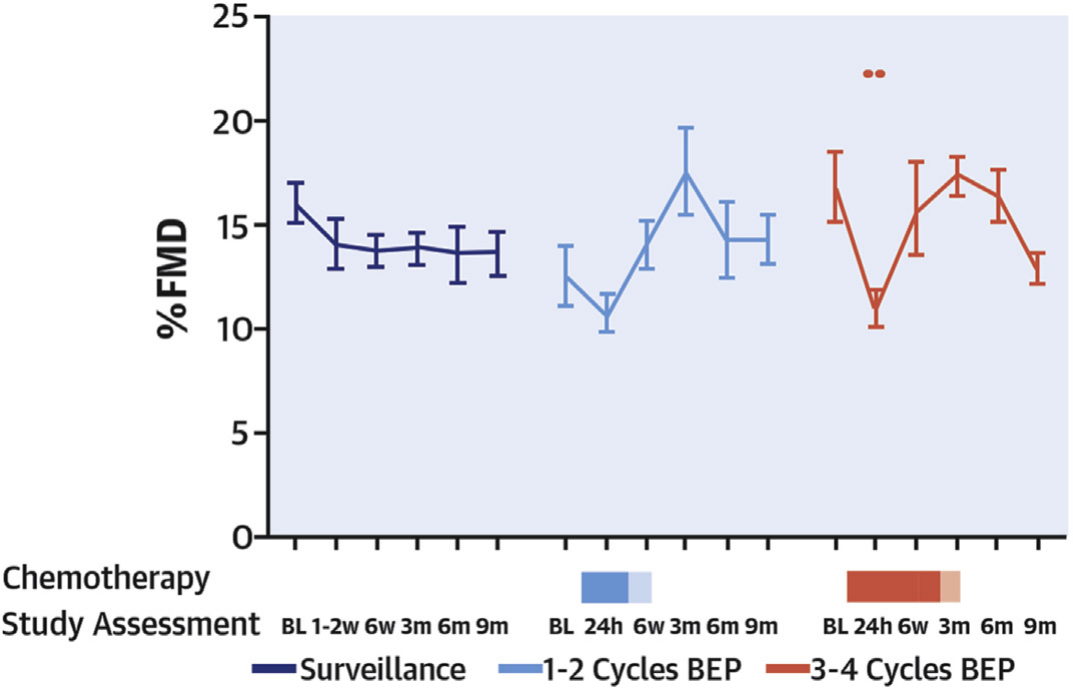
Cohort 1 (Early Effects Study): %FMD Score Changes in %FMD among patients managed with surveillance **(dark blue line)**, 1 to 2 cycles bleomycin, etoposide, and cisplatin (BEP) **(light blue line)**, and 3 to 4 cycles BEP **(red line)**. The **blue and red shaded rectangles** refer to duration of chemotherapy treatment (**solid blue** = 1 cycle, **light blue** = 2 cycles; **solid red** = 3 cycles, **light red** = 4 cycles). **Shaded areas** indicate chemotherapy. ∗∗p < 0.01. BL = baseline; FMD = flow-mediated dilatation; m = month; W = week.

Participant characteristics are presented in Table 1. %FMD decreased 24 h after the first dose of cisplatin in the 10 patients managed with 3 to 4 cycles of BEP (16.7 ± 1.6 at baseline vs. 10.9 ± 0.9 at 24 h after cisplatin; p = 0.003) (Figure 2). At 6 weeks, %FMD had returned to baseline (15.7 ± 2.1; p = 0.97 vs. baseline) (Figure 2, Supplemental Table 1). %FMD was unchanged compared with baseline at all other times (Figure 2) (all p > 0.05). In the 7 patients managed with 1 to 2 cycles of BEP or surveillance, %FMD was not significantly different from baseline at any point (Figure 2) (p > 0.05). %FMD data were available for 157 of 162 study visits. Resting BP and HR were unchanged during treatment and follow-up in all groups (p = NS for all; data not shown).

**Table 1:** Cohort 1 (Early Effects Study): Baseline Characteristics in Groups Treated With Different Chemotherapy Strategies

	Surveillance (n = 10)	1 to 2 Cycles of BEP Low-Intensity Cisplatin (n = 7)	3 to 4 Cycles of BEP High-Intensity Cisplatin (n = 10)
Age, yrs	39 ± 3	31 ± 2	34 ± 2
Height, m	1.79 ± 0.02	1.78 ± 0.03	1.77 ± 0.03
Weight, kg	93.5 ± 7.0	90.4 ± 5.8	88.7 ± 3.8
Body mass index, kg/m^2^	28.9 ± 2.0	28.3 ± 1.4	28.3 ± 1.1
Systolic blood pressure, mm Hg	122.9 ± 5.3	134.1 ± 3.4	131.7 ± 4.3
Diastolic blood pressure, mm Hg	73.1 ± 4.2	76.7 ± 2.9	79.0 ± 3.0
Heart rate, beats/min	66.7 ± 3.0	70.9 ± 5.3	64.7 ± 2.8
Cholesterol, mmol/l	5.2 ± 0.3	5.1 ± 0.5	5.5 ± 0.2
Triglycerides, mmol/l	1.2 ± 0.2	1.1 ± 0.2	1.9 ± 0.5
LDL cholesterol, mmol/l	3.3 ± 0.3	3.4 ± 0.4	3.5 ± 0.2
HDL cholesterol, mmol/l	1.2 ± 0.1	1.3 ± 0.1	1.8 ± 0.4
Glucose, mmol/l	4.9 ± 0.1	5.1 ± 0.2	5.0 ± 0.2
HbA1c, mmol/mol	34.7 ± 1.1	33.1 ± 1.0	35.5 ± 1.2
vWF:Ag, %	44.3 ± 4.8	38.4 ± 3.4	45.2 ± 2.8
Log urine ACR, mg/l	−0.18 ± 0.10	−0.23 ± 0.08	−0.14 ± 0.05
Histological diagnosis			
Seminoma	7 (70)	—	2 (20)
Nonseminoma/mixed	3 (30)	7 (100)	8 (80)
Performance status			
0	10 (100)	7 (100)	6 (60)
1	—	—	4 (40)
Medical history			
Hypertension	1 (10)	—	1 (10)
Diabetes	-	—	—
Smoker	1 (10)	—	—
Medications			
Alpha-blocker	—	—	1 (10)
Angiotensin-II receptor blocker	—	—	1 (10)

Values are mean ± SEM or n (%). Units reported in mmol/l can be converted to mg/dl through the following conversion factors: cholesterol mmol/l = mg/dl ÷ 38.6; triglyceride mmol/l = mg/dl ÷ 88.5; Glucose mmol/l = mg/dl ÷ 18.

ACR = albumin to creatinine ratio; BEP = bleomycin, etoposide, and cisplatin; HDL = high-density lipoprotein; LDL = low-density lipoprotein; vWF:Ag = von Willebrand factor antigen.

#### Cardiovascular biomarkers

In patients managed with 3 to 4 cycles of BEP, serum cholesterol increased at 6 weeks (7.2 ± 0.5 mmol/l vs. 5.5 ± 0.2 mmol/l at baseline; p = 0.012) (Table 2). This remained numerically greater than baseline thereafter, but was not statistically significant. There were trends toward increased triglycerides and low-density lipoprotein (LDL) cholesterol in patients managed with 3 to 4 cycles of BEP (p = 0.076 and p = 0.079 vs. baseline). This pattern was not seen in the other groups. Serum fasting glucose increased within 24 h in patients managed with 1 to 2 or 3 to 4 cycles of BEP (both p < 0.0001 vs. baseline), but did not change in patients managed with surveillance. There was a rise in HbA1c in the patients receiving 3 to 4 cycles of BEP at 6 weeks (p < 0.001), but thereafter it was not elevated versus baseline. vWF antigen increased at 6 weeks versus baseline in the 3 to 4 cycles of BEP group (p = 0.048) but was not different from baseline at any other time point or in any other group. High sensitivity troponin-I, ICAM-1, and high-sensitivity C-reactive protein were unchanged throughout (Table 2).

#### Renal biomarkers

**Figure 3:**
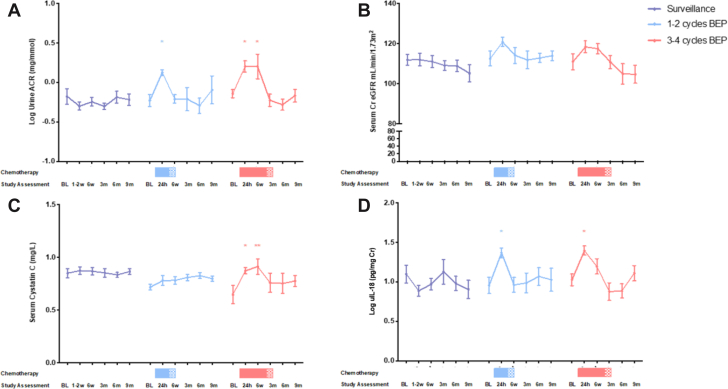
Cohort 1 (Early Effects Study): Renal Biomarkers Changes in renal biomarkers among patients managed with surveillance **(purple line),** 1 to 2 cycles BEP **(blue line)**, and 3 to 4 cycles BEP **(red line)**: **(A)** Log urine ACR; **(B)** eGFR; **(C)** serum cystatin C; **(D)** log urine IL-18 per urine creatinine. **Shaded areas** indicate chemotherapy. The p values represent 2-way analysis of variance with correction for multiple comparisons. ∗p < 0.05; ∗∗p < 0.01; ∗∗∗p < 0.001. ACR = albumin to creatinine ratio; Cr = creatinine; eGFR = estimated glomerular filtration rate; IL = interleukin; other abbreviations as in Figure 2.

In patients managed with 3 to 4 cycles of BEP, urine ACR increased at 24 h and 6 weeks (p = 0.011 and p = 0.014) and returned to baseline thereafter (Figure 3). Urine IL-18 increased at 24 h (p = 0.023) and returned to baseline by 6 weeks. Serum cystatin C increased at 24 h (p = 0.012) and 6 weeks (p = 0.004) and returned to baseline thereafter. eGFR was unchanged throughout (Figure 3). Urine ACR and IL-18 increased 24 h after 1 to 2 cycles of BEP (p = 0.038 and p = 0.039) and returned to baseline by 6 weeks. There were no other significant changes in this group or patients managed with surveillance.

### Cohort 2: medium-term effects of cisplatin-based chemotherapy

#### Forearm arterial vasomotor function

**Figure 4:**
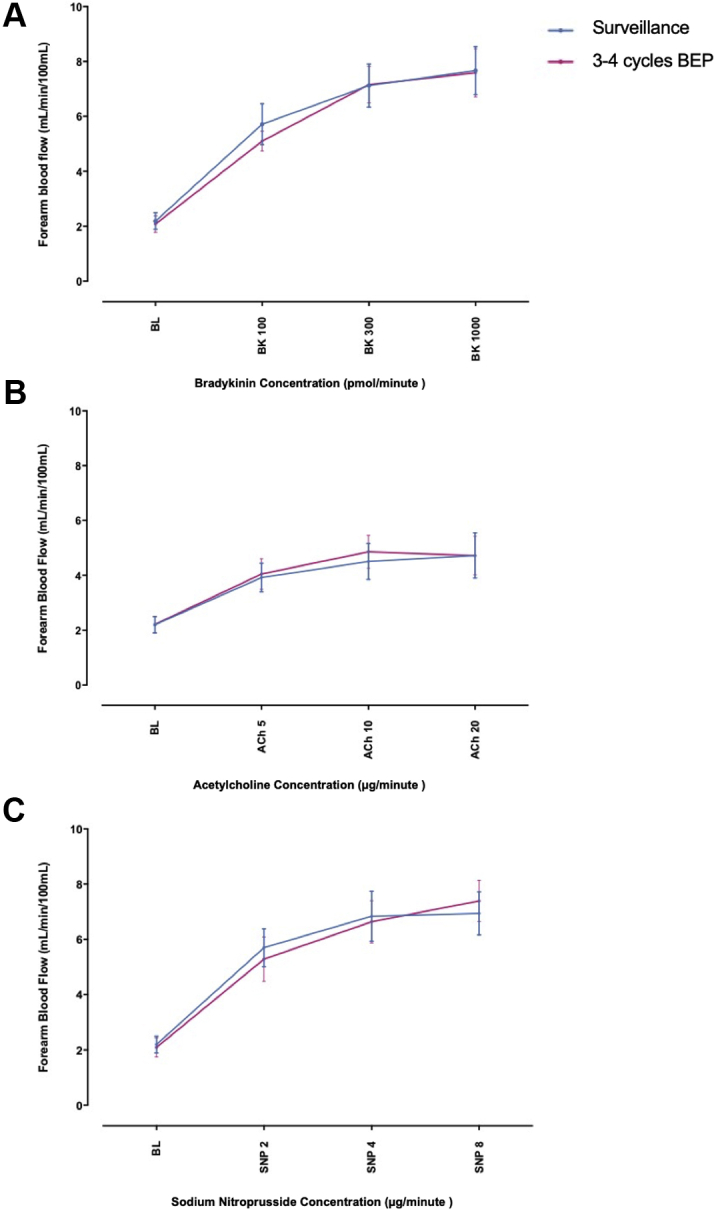
Cohort 2 (Medium-Term Effects Study): Absolute Forearm Blood Flow Changes in absolute forearm blood flow among patients managed 1 to 7 years previously with orchidectomy plus 3 to 4 cycles BEP **(red line)** versus orchidectomy alone **(blue line)**: **(A)** bradykinin 100, 300, and 1,000 pmol/min; **(B)** acetylcholine 5, 10, and 20 μg/min; **(C)** sodium nitroprusside 2, 4, and 8 μg/min. The p values represent 2-way analysis of variance with correction for multiple comparisons. ACh = acetylcholine; BK = bradykinin; SNP = sodium nitroprusside; other abbreviations as in Figure 2.

Baseline characteristics are presented in Table 3. Intra-arterial BK, ACh, and SNP evoked dose-dependent vasodilation in all participants (all p < 0.0001) (Figure 4). Vasodilator responses to BK, ACh, and SNP were not different in patients managed with 3 to 4 cycles of BEP (n = 12) versus surveillance (n = 14) (p = 0.811, 0.866, and 0.938, respectively (Figure 4, Supplemental Table 2). BK-induced vasodilation data were available in 11 of 12 subjects managed with 3 to 4 cycles of BEP and all 14 subjects (100%) managed with surveillance.

**Table 2:** Cohort 1 (Early Effect): Cardiovascular Biomarkers

	Chol (mmol/l)	HDL-C (mmol/l)	LDL-C (mmol/l)	TGs (mmol/l)	Gluc (mmol/l)	HbA1C (mmol/mol)	vWF:Ag (%)	t-PA (ng/ml)	PAI-1 (ng/ml)	hs-CRP (mg/l)	ICAM-1 (ng/ml)	hs-TNI (pg/ml)	Log Urine ACR (mg/l)	
Surveillance (n = 10)														
BL	5.2 ± 0.3	1.2 ± 0.1	3.3 ± 0.3	1.2 ± 0.2	4.9 ± 0.1	34.7 ± 1.1	44.3 ± 4.8	11.9 ± 2.5	111.2 ± 4.3	2.1 ± 1.0	408.1 ± 37.4	0.24 ± 0.16	−0.18 ± 0.10	
1–2 weeks	5.1 ± 0.3	1.3 ± 0.1	3.2 ± 0.2	1.1 ± 0.2	4.9 ± 0.1	34.2 ± 0.9	42.8 ± 5.2	13.5 ± 3.1	116.7 ± 3.3	2.1 ± 0.7	389.3 ± 47.7	0.19 ± 0.19	−0.30 ± 0.05	
6 weeks	5.0 ± 0.3	1.2 ± 0.1	3.2 ± 0.2	1.2 ± 0.1	5.0 ± 0.2	33.4 ± 0.8	45.9 ± 5.3	12.1 ± 2.4	113.0 ± 3.1	1.7 ± 0.7	387.6 ± 39.1	0.47 ± 0.35	−0.25 ± 0.06	
3 months	4.7 ± 0.3	1.2 ± 0.1	3.0 ± 0.2	1.1 ± 0.1	5.1 ± 0.2	33.8 ± 0.7	43.2 ± 4.1	12.8 ± 2.5	111.3 ± 5.3	2.0 ± 0.8	417.4 ± 32.1	0.13 ± 0.13	−0.30 ± 0.04	
6 months	4.7 ± 0.3	1.2 ± 0.1	2.9 ± 0.2	1.2 ± 0.2	5.1 ± 0.2	33.8 ± 0.5	48.2 ± 4.6	13.7 ± 2.6	110.9 ± 3.9	3.4 ± 1.8	454.9 ± 58.9	0.15 ± 0.11	−0.18 ± 0.07	
9 months	4.8 ± 0.4	1.2 ± 0.1	3.0 ± 0.2	1.2 ± 0.2	5.0 ± 0.2	35.0 ± 1.0	51.2 ± 6.7	16.7 ± 2.4	101.8 ± 6.2	1.8 ± 0.8	460.4 ± 23.4	0.31 ± 0.21	−0.22 ± 0.07	
1–2 cycles of BEP (low-intensity cisplatin) (n = 7)														
BL	5.1 ± 0.5	1.3 ± 0.1	3.4 ± 0.4	1.1 ± 0.2	5.1 ± 0.2	33.1 ± 1.0	38.4 ± 3.4	9.0 ± 1.2	105.6 ± 8.0	3.8 ± 1.5	353.6 ± 37.5	0.19 ± 0.19	−0.23 ± 0.08	
24 h	4.9 ± 0.4	1.3 ± 0.1	3.3 ± 0.4	0.7 ± 0.1	6.9 ± 0.2∗	33.7 ± 0.9	43.1 ± 1.6	6.1 ± 0.9	108.9 ± 6.7	2.2 ± 0.5	355.1 ± 32.6	0.43 ± 0.29	0.13 ± 0.04†	
6 weeks	5.8 ± 0.8	1.2 ± 0.1	3.8 ± 0.7	2.1 ± 0.3	5.1 ± 0.2	35.6 ± 1.1	52.8 ± 1.9	7.2 ± 0.9	109.5 ± 2.8	4.4 ± 1.9	437.8 ± 57.7	1.21 ± 0.50	−0.21 ± 0.05	
3 months	5.1 ± 0.8	1.2 ± 0.1	3.4 ± 0.7	1.5 ± 0.3	5.0 ± 0.2	30.0 ± 0.7	44.3 ± 6.0	10.5 ± 2.4	111.1 ± 3.7	3.4 ± 1.3	436.9 ± 49.7	0.24 ± 0.24	−0.21 ± 0.15	
6 months	5.2 ± 0.5	1.2 ± 0.1	3.4 ± 0.4	1.4 ± 0.3	4.9 ± 0.1	35.0 ± 1.1	55.8 ± 8.4	15.4 ± 3.6	105.0 ± 5.9	1.6 ± 0.5	452.7 ± 41.3	0.94 ± 0.51	−0.29 ± 0.10	
9 months	4.9 ± 0.4	1.2 ± 0.1	3.2 ± 0.3	1.2 ± 0.1	5.2 ± 0.2	32.7 ± 0.9	55.0 ± 8.2	13.4 ± 3.2	101.7 ± 8.0	4.0 ± 2.1	425.6 ± 45.0	0.27 ± 0.27	−0.09 ± 0.18	
3–4 cycles of BEP (high-intensity cisplatin) (n = 10)														
BL	5.5 ± 0.2	1.8 ± 0.4	3.5 ± 0.2	1.9 ± 0.5	5.0 ± 0.2	35.5 ± 1.2	45.2 ± 2.8	13.4 ± 1.3	114.4 ± 2.8	2.7 ± 1.5	411.1 ± 28.9	0.83 ± 0.55	−0.14 ± 0.05†	
24 h	5.7 ± 0.3	1.8 ± 0.4	4.0 ± 0.3	1.1 ± 0.2	6.6 ± 0.2∗	35.3 ± 1.3	42.4 ± 2.7	13.5 ± 1.3	110.7 ± 2.9	1.4 ± 0.5	382.2 ± 32.1	0.12 ± 0.12	0.20 ± 0.07†	
6 weeks	7.2 ± 0.5†	2.3 ± 0.6	4.7 ± 0.5	2.6 ± 0.5	5.2 ± 0.3	41.8 ± 2.0∗	62.4 ± 5.4†	14.6 ± 2.7	115.5 ± 3.7	3.1 ± 1.6	460.8 ± 43.1	1.39 ± 0.63	0.20 ± 0.16	
3 months	6.7 ± 0.4	1.8 ± 0.4	4.2 ± 0.4	3.1 ± 0.7	5.0 ± 0.1	29.1 ± 1.7∗	48.9 ± 3.8	15.1 ± 2.0	104.5 ± 3.5	3.7 ± 1.9	495.2 ± 20.5	1.41 ± 0.70	−0.23 ± 0.08	
6 months	6.2 ± 0.4	1.7 ± 0.4	3.8 ± 0.3	2.9 ± 0.5	5.1 ± 0.2	37.8 ± 0.9	47.7 ± 4.9	16.1 ± 2.6	108.0 ± 3.2	1.5 ± 0.4	392.2 ± 62.4	0.47 ± 0.24	−0.28 ± 0.07	
9 months	6.1 ± 0.3	1.8 ± 0.5	4.1 ± 0.3	2.4 ± 0.3	5.1 ± 0.2	33.8 ± 0.9	48.2 ± 3.0	15.3 ± 2.5	110.4 ± 3.9	3.4 ± 1.5	440.8 ± 40.1	1.22 ± 0.40	−0.17 ± 0.08	

Values are mean ± SEM.

BL = baseline; Chol = cholesterol; HDL-C = high-density lipoprotein cholesterol; LDL-C = low-density lipoprotein cholesterol; gluc = glucose; hs-CRP = high-sensitivity C-reactive protein; hs-TNI = high-sensitivity troponin I; ICAM = intracellular adhesion molecule; PAI = plasminogen activator inhibitor; TG = triglyceride; t-PA = tissue plasminogen activator; vWF:Ag = von Willebrand factor antigen.

∗ p < 0.001;

† p < 0.05.

**Table 3:** Cohort 2 (Medium-Term Effects): Baseline Characteristics in Groups Treated With Different Chemotherapy Strategies

	Surveillance (n = 14)	3 to 4 Cycles of BEP (n = 12)
Age, yrs	38 ± 2	36 ± 2
Body mass index, kg/m^2^	25.6 ± 0.9	26.9 ± 0.8
Systolic blood pressure, mm Hg	122.6 ± 3.7	128.4 ± 3.1
Diastolic blood pressure, mm Hg	74.9 ± 2.7	74.1 ± 2.7
Heart rate, beats/min	60.1 ± 2.5	60.9 ± 3.3
Cholesterol, mmol/l	5.1 ± 0.3	4.9 ± 0.3
Triglycerides, mmol/l	1.2 ± 0.2	1.4 ± 0.2
LDL cholesterol, mmol/l	3.2 ± 0.3	3.1 ± 0.2
HDL cholesterol, mmol/l	1.4 ± 0.1	1.3 ± 0.1
Glucose, mmol/l	4.7 ± 0.1	4.5 ± 0.4
HbA1c, mmol/mol	32.7 ± 0.7	33.1 ± 0.8
vWF:Ag, %	40.1 ± 2.2	43.0 ± 1.1
Histological diagnosis		
Seminoma	8 (57)	2 (17)
Nonseminoma/mixed	6 (43)	10 (83)
Performance status		
0	14 (100)	12 (100)

Values are mean ± SEM or n (%). Units reported in mmol/l can be converted to mg/dl through the following conversion factors: cholesterol mmol/l = mg/dl ÷ 38.6; triglyceride mmol/l = mg/dl ÷ 88.5; glucose mmol/l = mg/dl ÷ 18.

Abbreviations as in Table 2.

#### BK-induced release of fibrinolytic factors

BK evoked dose-dependent net t-PA antigen release in patients managed with surveillance (−1.8 ± 10.6 ng/100 ml/min [baseline] vs. 316.4 ± 57.3 ng/100 ml/min [BK 1,000 ng/min]; p < 0.001) and 3 to 4 cycles of BEP (−7.7 ± 7.7 ng/100 ml/min [baseline] vs. 263.9 ± 67.7 ng/100 ml/min [BK 1,000 ng/min]; p < 0.001). There was no differences between groups (p = 0.285). BK did not evoke changes in net PAI-1 antigen in patients managed with surveillance (p = 0.524) or 3 to 4 cycles BEP (p = 0.502) and responses were not different between groups (p = 0.946).

### In vitro effects of cisplatin on thrombotic and stress kinase pathways

**Figure 5:**
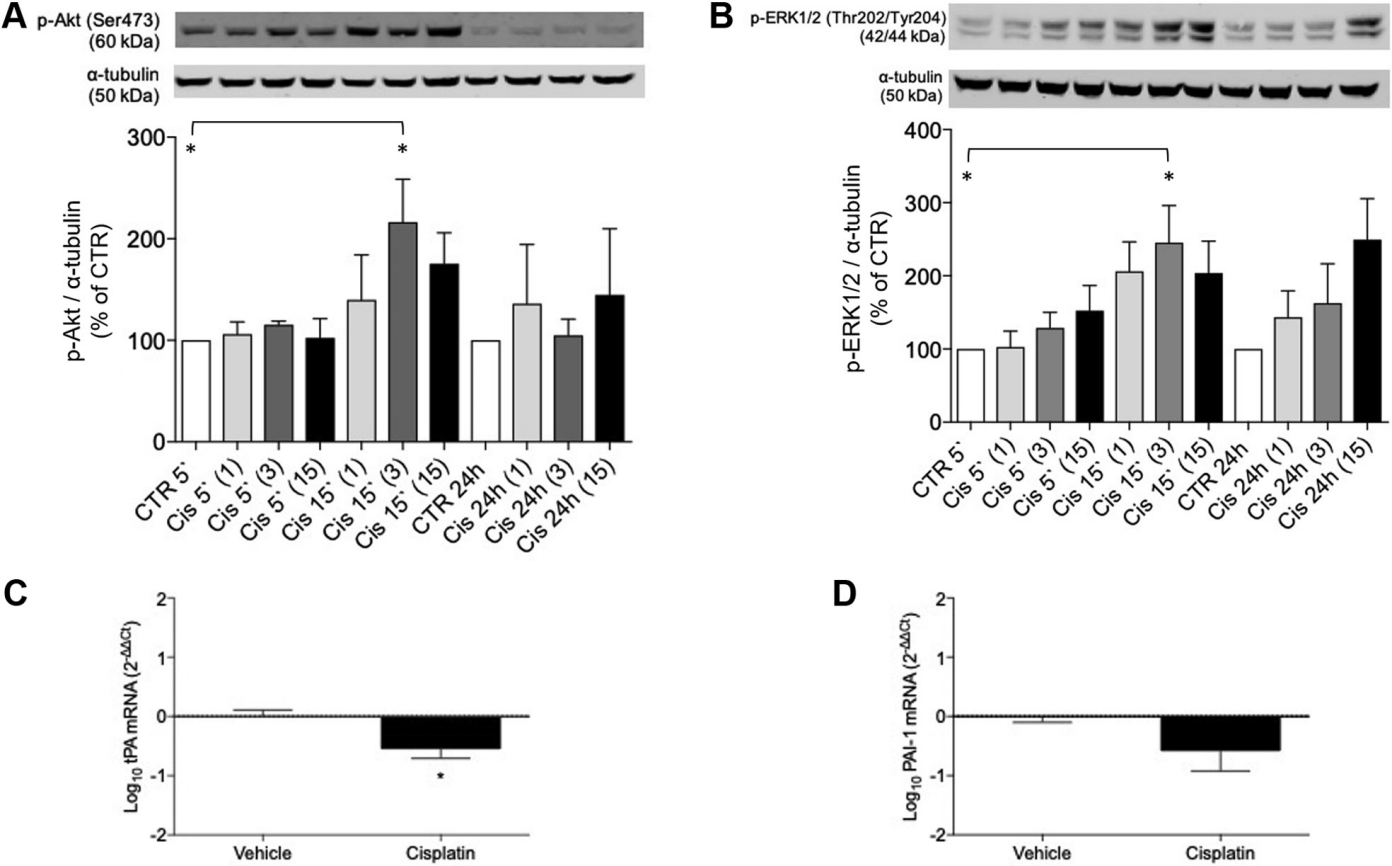
In Vitro Effects From Cisplatin on Stress Kinase Signaling and Thrombotic Pathways in Human Aortic Endothelial Cells Effects from cisplatin on phosphorylation of Akt **(A)** and ERK1/2 **(B)**, and mRNA expression of t-PA **(C)** and PAI-1 **(D)**. The p values represent 1-way analysis of variance with correction for multiple comparisons (Western Blot analyses) or unpaired Student’s *t*-test (mRNA expression). ∗p < 0.05. 5ʹ = 5 min; 15ʹ = 15 min; (1) = cisplatin 1 μg/ml; (3) = cisplatin 3 μg/ml; (15) = cisplatin 15 μg/ml; Cis = cisplatin; CTR = control; PAI = plasminogen activator inhibitor; t-PA = tissue plasminogen activator.

HAEC exposure to cisplatin 3 μg/ml for 15 min increased Akt phosphorylation compared with control (n = 5; p = 0.032) (Figure 5). No changes were observed with other concentrations or periods of cisplatin exposure. Similar results were found for ERK 1/2 phosphorylation (n = 5; p = 0.026). t-PA mRNA expression decreased in cells exposed to cisplatin (n = 7; p = 0.014), whereas PAI-1 mRNA expression was unchanged (n = 7; p = 0.122) (Figure 5).

## Discussion

Cisplatin-based chemotherapy is associated with endothelial vasomotor dysfunction, hypercholesterolemia, hyperglycemia, and renal dysfunction in testicular cancer patients (Central Illustration). These effects are confined to the immediate perichemotherapy period. Our observations suggest the early time period post-cisplatin treatment is one that is potentially of increased renal and cardiovascular risk, and one during which attention should be given to risk reduction with cardiovascular protection strategies.

**Central Illustration:**
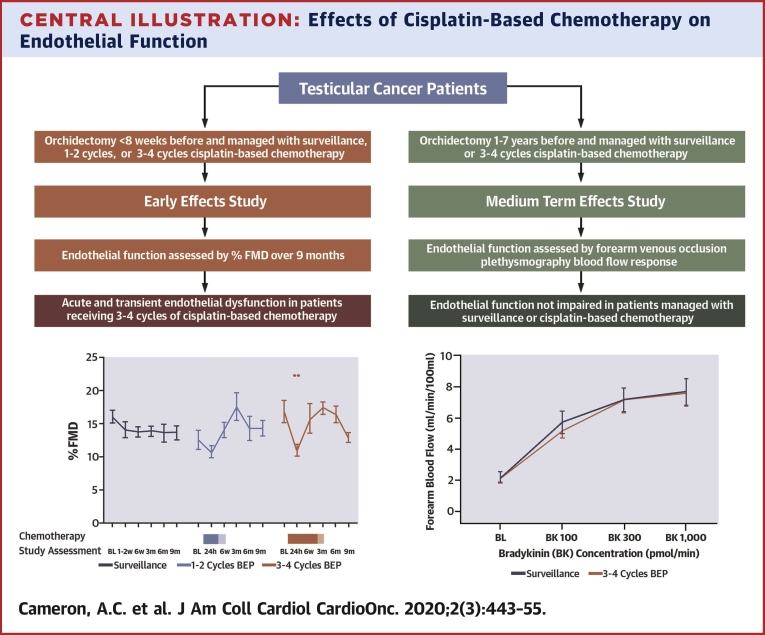
Effects of Cisplatin-Based Chemotherapy on Endothelial Function Patients with testicular cancer managed with cisplatin-based chemotherapy or surveillance were enrolled. Endothelial function was assessed by % flow-mediated dilatation over 9 months in patients with a recent diagnosis, or venous occlusion plethysmography in patients managed 1 to 7 years previously. Acute and transient endothelial dysfunction was observed 24 h after cisplatin-based chemotherapy, and endothelial function was not impaired 1 to 7 years after treatment. In the **left panel**, the **blue and red shaded rectangles** refer to duration of chemotherapy treatment (**solid blue** = 1 cycle, **light blue** = 2 cycles; **solid red** = 3 cycles, **light red** 4 = cycles). **Shaded areas** indicate chemotherapy. ∗∗p < 0.01.

A strength of the study is the early assessment of endothelial vasomotor function within 24 h following chemotherapy. Prior studies evaluating vascular function after cisplatin-based chemotherapy are limited by infrequent assessments performed months or years following treatment (6,9), potentially missing the period of maximum vascular injury and dysfunction. We demonstrate that, following 3 to 4 cycles of BEP, endothelial function is impaired at this acute and vulnerable time. Although endothelial function returned to baseline values at the subsequent 6-week assessment, it is biologically plausible that a similar transient deterioration in endothelial function is induced with each treatment cycle (5). Endothelial dysfunction is a key feature in de novo thrombosis and rupture of pre-existing atherosclerotic plaque (25). As such, it is an important component to the risk of thrombotic cardiovascular events in the early period following cisplatin-based chemotherapy.

In our cell-based study, exposure of HAECs to cisplatin was associated with decreased t-PA mRNA expression and activation of Akt and ERK 1/2. In previous work, cisplatin has been shown to reduce endothelial cell survival and induce apoptosis in human dermal microvascular endothelial cells (HMEC-1) (26) and in the vasa nervorum in rats (27). These data support the hypothesis that cisplatin induces direct endothelial toxic effects resulting in increased stress kinase signaling and a propensity for thrombosis via reduction in the capacity for endogenous fibrinolysis.

Although adverse thrombotic effects are most frequently observed in the early period following chemotherapy, cisplatin is detectable in serum for several years after treatment (28). This chronic exposure may provoke low-grade endothelial stimulation and a consequent pro-atherogenic environment (28,29). Our subsequent assessment was with forearm venous occlusion plethysmography, the gold standard for assessing endothelial function (12). In patients treated 1 to 7 years previously, there was no difference in endothelial vasomotor function between patients managed with orchidectomy and cisplatin-based chemotherapy versus those managed with orchidectomy alone. Furthermore, BK-induced t-PA release was not different in patients managed with or without cisplatin-based chemotherapy. The capacity for endothelial t-PA release is a sensitive, and mechanistically relevant, marker for the prediction of patients at risk of cardiovascular events (30).

Serum cholesterol and HbA1C increased transiently at 6 weeks after 3 to 4 cycles of BEP and similar trends were seen for LDL cholesterol and triglycerides. Although cisplatin-based chemotherapy may have contributed to these observations, we cannot conclude that this is the only explanation. Indeed, a reduction in tumor burden may have contributed to increases in cholesterol (31). Furthermore, glucocorticoid antiemetics may have contributed to changes in lipid profile and glycemia. However, we do not believe glucocorticoids are the sole explanation for the change in lipid parameters. Indeed, in a randomized study in which dexamethasone or placebo were given to healthy male volunteers, dexamethasone did not affect endothelial function, triglycerides, or LDL cholesterol, albeit with a different dexamethasone regimen to our study (32).

Most studies evaluating the risk of CVD after cisplatin-based chemotherapy have focused on the later period after treatment (3,8,33,34). In the 10 to 19 years following treatment, a 5- to 7-fold increased risk of CVD has been reported compared with patients managed with surveillance or the general population (8,33). Larger studies of up to 2,700 patients demonstrate a more modest 1.5- to 2-fold increased risk of CVD at 10 to 18 years after treatment (3,34). However, the incidence of thrombosis appears to be highest early after treatment. Between 9% and 11% have a thrombotic event within 1 year following 3 or more cycles of cisplatin-based chemotherapy (9,35). Importantly, a more granular assessment of 15,006 patients revealed a 5-fold increased risk of cardiovascular death in the first year after treatment. This risk fell dramatically thereafter and was not significant after 1 year (1). Therefore, in keeping with our observations, the early period after treatment with 3 or more cycles of cisplatin-based chemotherapy appears to be the time period of increased cardiovascular risk. While these effects predominantly occur in the early period after treatment, cisplatin is detectable in serum several years after treatment (28). It remains possible that chronic exposure to low levels of cisplatin may cause low-grade endothelial stimulation that contributes to the pathophysiology underlying cardiovascular events occurring more than a decade after initial exposure (8,28).

Nephrotoxicity is a major, dose-limiting side effect of cisplatin that affects 20% to 40% of patients (4,36). We found evidence of early nephrotoxicity following 3 to 4 cycles of BEP as illustrated by increased serum cystatin C (37). Importantly, this returned to baseline by 3 months and, although there was an absolute decrease in eGFR, the change in this less-sensitive measure did not reach statistical significance. Increased urinary ACR in the immediate period following chemotherapy supports the hypothesis that cisplatin induces renovascular endothelial and proximal tubular dysfunction and inflammation as evidenced by the rise in urinary IL-18 (4,36,38). We hypothesize that this profile of renal injury is compatible with widespread endothelial activation and injury.

### Study limitations

To our knowledge this is one of the most comprehensive, longitudinal evaluations of changes in vascular and renal function after cisplatin-based chemotherapy. However, there are some limitations that should be acknowledged. The number of patients in cohort 1 is small, particularly the group managed with 1 to 2 cycles of BEP, and it is not possible to conclude whether associations in this group did not reach statistical significance because of reduced power or because greater cancer burden potentiates the toxic effect of cisplatin-based chemotherapy. Furthermore, we did not adjust for potential confounders that may have contributed, such as the baseline elevated BMI in all groups, acute hyperglycemia perhaps due to steroids, and an acute inflammatory response to tumor cell death.

Vascular function was assessed using different approaches in the acute and long-term studies. The use of portable equipment allowed assessment of endothelial function at the patient’s bedside in the immediate post-chemotherapy period. We reserved the use of forearm venous occlusion plethysmography for studies in survivors after 1 year. These techniques primarily examine function at different levels of the arterial tree, and we accept this as a limitation. We did not use forearm venous occlusion plethysmography to assess the early vascular effects of cisplatin because this requires arterial cannulation in the context of a potentially pro-thrombotic state and is impractical in the peri-chemotherapy period. Serial assessment of %FMD each day during chemotherapy provides insight to the duration of effects from cisplatin on endothelial function; it would be helpful to assess %FMD in the period at least 1 year after treatment, but we limited assessments to maximize recruitment and retention of patients undergoing intensive treatment regimes. Although AngioDefender %FMD correlates with Framingham risk score (14), it has not been demonstrated that AngioDefender %FMD is independently predictive of cardiovascular risk. Although we demonstrate deleterious effects of cisplatin-based chemotherapy in the early phase that are not evident in the medium-term, these findings could be further strengthened by the inclusion of a larger number of participants and the assessment of survivors treated >7 years previously. Heightened risk of CVD is reported up to 20 years after cisplatin-based chemotherapy (8), although endothelial function has not been assessed beyond 7 years after treatment, and it remains possible that endothelial dysfunction is also detectable in longer-term survivors.

## Conclusions

Acute and transient endothelial toxicity, dyslipidemia, hyperglycemia, and nephrotoxicity are apparent in the early period following cisplatin-based chemotherapy, when cardiovascular risk is greatest (1). Our data highlight the early perichemotherapy period as an important window for focused surveillance of cardiovascular and renal health during which baseline and emergent cardiovascular risk factors should be treated aggressively. The evaluation of short-term preventative strategies, such as statins and antithrombotic therapies, is warranted in this group to allow cancer survivorship to come at the minimum cardiovascular cost.

Perspectives **COMPETENCY IN MEDICAL KNOWLEDGE:** Cisplatin-based chemotherapy is associated with acute and transient endothelial dysfunction, dyslipidemia, hyperglycemia, and nephrotoxicity in patients with testicular cancer. Our findings suggest that the early period following cisplatin-based chemotherapy is an important time period where functional and biological perturbations in cardiovascular and renal function occur.

**TRANSLATIONAL OUTLOOK:** The early period following cisplatin-based chemotherapy may be an important window for surveillance and aggressive management of cardiovascular and renal health. Early preventive strategies, including those with statins and antithrombotic therapies, should be further investigated in patients treated with cisplatin-based chemotherapy to mitigate cardiovascular risk in the long-term.

## References

[1] Fung C., Fossa S.D., Milano M.T. (2015). Cardiovascular disease mortality after chemotherapy or surgery for testicular nonseminoma: a population-based study. J Clin Oncol.

[2] Hanna N., Einhorn L.H. (2014). Testicular cancer: a reflection on 50 years of discovery. J Clin Oncol.

[3] van den Belt-Dusebout A.W., Nuver J., de Wit R. (2006). Long-term risk of cardiovascular disease in 5-year survivors of testicular cancer. J Clin Oncol.

[4] Miller R.P., Tadagavadi R.K., Ramesh G., Reeves W.B. (2010). Mechanisms of cisplatin nephrotoxicity. Toxins.

[5] Soultati A., Mountzios G., Avgerinou C. (2012). Endothelial vascular toxicity from chemotherapeutic agents: Preclinical evidence and clinical implications. Cancer Treat Rev.

[6] Nuver J., Smit A.J., Sleijfer D.T. (2004). Microalbuminuria, decreased fibrinolysis, and inflammation as early signs of atherosclerosis in long-term survivors of disseminated testicular cancer. Eur J Cancer.

[7] de Haas E.C., Altena R., Boezen H.M. (2013). Early development of the metabolic syndrome after chemotherapy for testicular cancer. Ann Oncol.

[8] Haugnes H.S., Wethal T., Aass N. (2010). Cardiovascular risk factors and morbidity in long-term survivors of testicular cancer: a 20-year follow-up study. J Clin Oncol.

[9] Nuver J., Smit A.J., van der Meer J. (2005). Acute chemotherapy-induced cardiovascular changes in patients with testicular cancer. J Clin Oncol.

[10] Nuver J., Smit A.J., Wolffenbuttel B.H. (2005). The metabolic syndrome and disturbances in hormone levels in long-term survivors of disseminated testicular cancer. J Clin Oncol.

[11] Watanabe A., Tanabe A., Maruoka R. (2014). Fibrates protect against vascular endothelial dysfunction induced by paclitaxel and carboplatin chemotherapy for cancer patients: a pilot study. Int J Clin Oncol.

[12] Wilkinson I.B., Webb D.J. (2001). Venous occlusion plethysmography in cardiovascular research: methodology and clinical applications. Br J Clin Pharmacol.

[13] Garg N., Krishan P., Syngle A. (2015). Rosuvastatin improves endothelial dysfunction in ankylosing spondylitis. Clin Rheumatol.

[14] Everist Health AngioDefender white paper. https://angiodefender.com/wp-content/uploads/sites/2/2018/12/3.1-AngioDefender-White-Paper-1.pdf.

[15] Levey A.S., Stevens L.A., Schmid C.H. (2009). A new equation to estimate glomerular filtration rate. Ann Intern Med.

[16] Roberts D.H., Tsao Y., Breckenridge A.M. (1986). The reproducibility of limb blood flow measurements in human volunteers at rest and after exercise by using mercury-in-Silastic strain gauge plethysmography under standardized conditions. Clin Sci.

[17] Alves-Lopes R., Neves K.B., Anagnostopoulou A. (2020). Crosstalk between vascular redox and calcium signaling in hypertension involves TRPM2 (Transient Receptor Potential Melastatin 2) cation channel. Hypertension.

[18] Newby D.E., Wright R.A., Ludlam C.A. (1997). An in vivo model for the assessment of acute fibrinolytic capacity of the endothelium. Thromb Haemost.

[19] Newby D.E., Witherow F.N., Wright R.A. (2002). Hypercholesterolaemia and lipid lowering treatment do not affect the acute endogenous fibrinolytic capacity in vivo. Heart.

[20] Lang N.N., Gudmundsdóttir I.J., Boon N.A. (2008). Marked impairment of protease-activated receptor type 1-mediated vasodilation and fibrinolysis in cigarette smokers: smoking, thrombin, and vascular responses in vivo. J Am Coll Cardiol.

[21] Newby D.E., Sciberras D.G., Mendel C.M. (1997). Intra-arterial substance P mediated vasodilatation in the human forearm: pharmacology, reproducibility and tolerability. Br J Clin Pharmacol.

[22] Gudmundsdottir I.J., Lang N.N., Boon N.A. (2008). Role of the endothelium in the vascular effects of the thrombin receptor (protease-activated receptor type 1) in humans. J Am Coll Cardiol.

[23] Japp A.G., Cruden N.L., Amer D.A.B. (2008). Vascular effects of apelin in vivo in man. J Am Coll Cardiol.

[24] Dawes M., Brett S.E., Chowienczyk P.J. (1999). The vasodilator action of nebivolol in forearm vasculature of subjects with essential hypertension. Br J Clin Pharm.

[25] Hunter A.L., Shah A.S., Langrish J.P. (2017). Fire simulation and cardiovascular health in firefighters. Circulation.

[26] Nuver J., De Haas E.C., Van Zweeden M. (2010). Vascular damage in testicular cancer patients: a study on endothelial activation by bleomycin and cisplatin in vitro. Oncol Rep.

[27] Kirchmair R., Walter D.H., Ii M. (2005). Antiangiogenesis mediates cisplatin-induced peripheral neuropathy. Circulation.

[28] Brouwers E.E., Huitema A.D., Beijnen J.H., Schellens J.H. (2008). Long-term platinum retention after treatment with cisplatin and oxaliplatin. BMC Clin Pharmacol.

[29] Haugnes H.S., Oldenburg J., Bremnes R.M. (2015). Pulmonary and cardiovascular toxicity in long-term testicular cancer survivors. Urol Oncol.

[30] Robinson S.D., Ludlam C.A., Boon N.A., Newby D.E. (2007). Endothelial fibrinolytic capacity predicts future adverse cardiovascular events in patients with coronary heart disease. Arterioscler Thromb Vasc Biol.

[31] Llaverias G., Danilo C., Mercier I. (2011). Role of cholesterol in the development and progression of breast cancer. Am J Pathol.

[32] Brotman D.J., Girod J.P., Garcia M.J. (2005). Effects of short-term glucocorticoids on cardiovascular biomarkers. J Clin Endocrinol Metab.

[33] Meinardi M.T., Gietema J.A., van der Graaf W.T. (2000). Cardiovascular morbidity in long-term survivors of metastatic testicular cancer. J Clin Oncol.

[34] van den Belt-Dusebout A.W., de Wit R., Gietema J.A. (2007). Treatment-specific risks of second malignancies and cardiovascular disease in 5-year survivors of testicular cancer. J Clin Oncol.

[35] Lubberts S., Boer H., Altena R. (2016). Vascular fingerprint and vascular damage markers associated with vascular events in testicular cancer patients during and after chemotherapy. Eur J Cancer.

[36] Volarevic V., Djokovic B., Jankovic M.G. (2019). Molecular mechanisms of cisplatin-induced nephrotoxicity: a balance on the knife edge between renoprotection and tumor toxicity. J Biomed Sci.

[37] Laterza O.F., Price C.P., Scott M.G. (2002). Cystatin C: an improved estimator of glomerular filtration rate?. Clin Chem.

[38] Ozkok A., Edelstein C.L. (2014). Pathophysiology of cisplatin-induced acute kidney injury. Biomed Res Int.

