# Supplementary material for: Comprehensive Characterization of the Vascular Effects of Cisplatin-Based Chemotherapy in Patients With Testicular Cancer
Source: JACC CardioOncol. 2020 Sep 15;2(3):443–55. doi: 10.1016/j.jaccao.2020.06.004 (PMC7539369; doi:10.1016/j.jaccao.2020.06.004)
Supplement: Supplemental Tables 1 and 2 [file mmc1.docx]

Supplemental Table 1. AngioDefender % FMD Scores in Cohort 1

|  | **AngioDefender % FMD Score** | | |
| --- | --- | --- | --- |
| **Time** | **Surveillance** | **1-2 Cycles BEP** | **3-4 Cycles BEP** |
| Baseline | 21.6 15.1 14.8 13.8 16.6 20.5 12.9 15.7 14.7 14.4 | 18.8 8.3 10.3 17.7 9.6  13.0 10 | 19.7 26.6 15.7 23.2 13.2 12.2 14.5 9.9 16.8 15.3 |
| 24 hours | 19.4 13.9 18.0 6.0 11.2 15.9 15.3 14.7 12.3 13.9 | 13.0 9.7 8.7 7.3 10.5  14.9 10.1 | 13.2 11.8 7.4 6.7 15.6 10.7 7.9 9.9 14.4 11.7 |
| 6 weeks | 13.1 12.2 15.3 14.3 12.2 18.8 10.4 12.8 14.1 14.2 | 11.8 16.6 13 16.7 16.9 13.4 9.3 | 14.4 28.2 10.5 10.8 15.5 9.9 19.5 16.9 |
| 3 months | 17.8 14.9 15.1 10.0 12.3 14.6 12.5 11.7 15.9 13.6 | 19.5 21.4 14.1 24.6 12.5 12.6 | 17.1 18.0 14.3 19.6 19.4 10.7 18.8 17.7 16.6 20.8 |
| 6 months | 12.3 10.3 11.2 11.6 12.3 12.5 22.8 16.2 13.4 | 14.3 14.4 10.7 23.9 9.6 10.2 16.9 | 13.1 19.0 14.4 18.8 12.3 25.3 17.4 15.3 14.5 14.3 |
| 9 months | 10.8 11.1 11.6 13.0 12.9 19.1 10.8 18.9 15.7 12.3 | 18.0 14 14.3 18.5 9.6 11.0 14.1 | 12.9 17.5 12.7 14.5 9.4 10.2 12.7 12.7 12.7 |
| FMD, Flow-mediated dilatation | | | |

Supplemental Table 2. Absolute Forearm Blood Flow Values in Cohort 2

| **Agent and Dose** | **Absolute Forearm Blood Flow (mL/min/100mL)** | |
| --- | --- | --- |
| **Bradykinin** | **3-4 Cycles BEP** | **Surveillance** |
| Baseline | 2.94 1.89 4.12 0.85 2.16 2.22 3.22 1.20 1.73 1.52 1.08 | 2.22 1.42 1.56 5.16 2.13 1.03 1.37 3.65 1.91 3.2 1.97 2.08 1.94 1.07 |
| BK 100 | 4.86 6.45 7.10 4.88 6.42 4.90 4.45 3.60 4.57 5.59 3.28 | 4.90 1.89 7.41 7.25 3.46 2.71 3.83 10.00 2.92 9.16 6.71 8.42 8.57 2.81 |
| BK 300 | 5.64 9.33 7.33 5.64 8.96 11.67 7.64 3.72 5.20 7.00 6.64 | 11.67 3.46 7.57 7.92 7.81 3.28 4.69 10.72 3.97 10.49 7.54 9.84 7.45 3.34 |
| BK 1000 | 6.22 10.96 9.1 4.15 9.61 13.02 7.64 3.44 6.10 7.64 5.62 | 13.02 4.33 9.80 8.67 4.99 4.70 5.95 14.13 4.09 11.00 8.27 6.43 6.45 5.57 |
| **Acetylcholine** |  |  |
| Baseline | 2.94 1.89 2.58 4.12 0.85 2.16 2.22 3.22 1.73 1.52 1.08 | 2.22 1.42 1.56 5.16 2.13 1.03 1.37 3.65 1.91 3.20 1.97 2.08 1.94 1.07 |
| ACh 5 | 4.02 4.68 5.76 4.77 0.97 7.61 3.94 3.46 5.02 2.07 2.25 | 3.94 3.44 5.93 3.47 3.50 1.56 1.28 8.10 2.51 5.77 5.63 3.63 4.64 1.49 |
| ACh 10 | 2.82 5.91 5.86 6.12 2.28 8.55 6.62 4.81 4.75 2.97 2.78 | 6.62 3.57 6.86 7.07 2.14 1.84 1.38 8.59 1.88 6.59 5.32 3.98 5.74 1.52 |
| ACh 20 | 3.43 6.29 6.29 3.83 2.39 8.16 8.00 6.25 2.76 2.88 1.63 | 8.00 4.06 5.36 7.37 1.46 1.14 0.85 11.48 2.63 4.78 4.31 6.86 6.30 1.52 |
| **Sodium Nitroprusside** |  |  |
| Baseline | 1.89 4.12 0.85 2.16 2.22 3.22 1.73 1.52 1.08 | 2.22 1.42 1.56 5.16 2.13 1.03 1.37 3.65 1.91 3.2 1.97 2.08 1.94 1.07 |
| SNP 2 | 7.80 6.15 1.10 7.18 6.12 7.33 1.91 6.05 3.90 | 6.30 3.81 5.64 7.46 3.92 3.87 3.27 11.6 3.29 9.07 6.89 7.63 3.48 3.57 |
| SNP 4 | 9.39 6.97 4.99 7.59 6.02 9.50 2.11 7.56 5.58 | 6.00 4.06 6.84 6.48 4.45 3.53 4.23 15.39 3.55 10.44 7.71 9.17 9.53 4.34 |
| SNP 8 | 10.99 7.65 6.01 6.95 5.40 9.84 3.99 9.16 6.56 | 5.70 2.67 9.65 3.45 5.77 4.64 4.85 10.73 5.47 10.52 9.07 9.74 10.46 4.46 |
| BK 100, bradykinin 100 pmol/minute; BK 300, bradykinin 300 pmol/minute; BK 1000, bradykinin 1000 pmol/minute.  ACh 5, acetylcholine 5 µg/minute; ACh 10, acetylcholine 10 µg/minute; ACh 20, acetylcholine 20 µg/minute.  SNP 2, sodium nitroprusside 2 µg/minute; SNP 4, sodium nitroprusside 4 µg/minute; SNP 8, sodium nitroprusside 8 µg/minute | | |
